# The Valorisation of Biochar Produced from Black Liquor Pyrolysis for the Development of CO_2_ Adsorbents

**DOI:** 10.3390/molecules29235613

**Published:** 2024-11-27

**Authors:** Anca Maria Zaharioiu, Violeta-Carolina Niculescu, Claudia Sandru, Stefan Ionut Spiridon, Amalia Soare, Simona Oancea, Florian Marin

**Affiliations:** 1 National Research and Development Institute for Cryogenic and Isotopic Technologies—ICSI Ramnicu Valcea, 4th Uzinei Street, 240050 Ramnicu Valcea, Romania; anca.zaharioiu@icsi.ro (A.M.Z.);; 2 Faculty of Agricultural Sciences, Food Industry and Environmental Protection, “Lucian Blaga” University of Sibiu, 7-9 I. Ratiu Str., 550012 Sibiu, Romania

**Keywords:** adsorbent, black liquor, biochar, catalysis, pyrolysis

## Abstract

The paper manufacturing process produces liquid and gaseous alternative fuels, as well as solid wastes. These can be subsequently treated through chemical processing, oxidation, and thermal activation, resulting in adsorbent materials with CO_2_ adsorption capacities. The valorisation of black liquor waste resulting from paper manufacturing was achieved through a catalytic pyrolysis process using two catalysts previously prepared in house (Cu-Zn-MCM-41 and Ni-SBA-16). The HCl-treated adsorbent material, resulting from Ni-SBA-16-catalysed pyrolysis, was selected for use in CO_2_ adsorption tests as it had the highest specific surface area (224.06 m^2^/g) and pore volume (0.28 cm^3^/g). The adsorption experimental setup was linked to a gas chromatograph in order to evaluate CO_2_ adsorption efficiency using a binary gas mixture consisting of 81% CO_2_ and 19% N_2_. With a CO_2_ adsorption capacity of 1.61 mmol/g, a separation efficiency of 99.78%, and a CO_2_ recovery yield of 90.02%, it can be concluded that the developed adsorbent material resulting from Ni-SBA16-catalysed pyrolysis and HCl treatment represents a viable solution for black liquor pyrolytic solid waste removal and reduction in greenhouse gases.

## 1. Introduction

One of the main causes of climate change is the increase in the concentration of greenhouse gases, which is due to the irrational consumption of fossil fuels in rising quantities. The main greenhouse gas is CO_2_. The emission of this gas causes major concerns and is responsible for the increase in global warming by more than 50% [1]. The concentration of CO_2_ in the atmosphere has increased rapidly, growing by over 100% compared to the base considered year—1850 [2]. Greenhouse gases are produced by various sectors, including industry, transport, and agricultural sector. This has led to the current CO_2_ concentration of approximately 400 ppm [3,4].

Global warming, the deterioration of the Earth’s climate, and the destruction of habitats have had a series of consequences, such as an increase in the temperature at the terrestrial level and an increase in the temperature at the level of the seas and oceans. These effects have caused the melting of glaciers, floods, fires, and extreme meteorological phenomena [5,6]. The global temperature may increase by 1.5 °C until 2030, and by 2 °C until 2050, as was predicted by the Intergovernmental Panel on Climate Change (IPCC). In 2015, the Paris Agreement, an international agreement signed by 196 countries making a legal commitment to reduce greenhouse gases, was sealed [7]. The Paris Agreement requires a significant reduction in CO_2_ concentrations in the atmosphere to prevent the global temperature from increasing. It proposes various measures and initiatives for the development of alternative, sustainable, and renewable fuels with increased energy efficiency to enable the replacement of fossil fuels, but also the capture of CO_2_ [8,9].

CO_2_ capture presents practical and economic advantages. CO_2_ capture can be achieved through various techniques, and it is possible to capture it before and after the combustion process. The process of CO_2_ capture and its conversion into value-added products has been promoted in recent years and is achieved through the development of functional materials such as adsorbents and catalysts [10,11,12,13]. 

Adsorbent materials developed from activated carbon were proved to be efficient for CO_2_ capture. This is due to their properties such as high specific surface area, increased adsorption capacity, and low costs. Carbon materials can be used in various fields of technology due to their properties such as resistance to wear and tear [14]. Over time, activated carbon was obtained from coal, wood and oil, but currently other raw materials are being investigated, such as industrial waste from sewage sludge, [15], bone meal [16] and black liquor, which is a waste from paper manufacturing. The preparation of adsorbent materials requires chemical, physical, and thermal activation to increase their adsorption capacity and the development of cavernous pores [17,18].

Black liquor (BL) is a waste resulting from the paper manufacturing processes. It is a complex viscous solution containing organic and inorganic materials. These are represented by lignin and residual alkaline salts [19,20]. In this study, black liquor waste was subjected to a pyrolysis process carried out at two temperatures, 300 °C and 450 °C, in an inert nitrogen atmosphere and in the absence of oxygen. This process was performed in the presence of bimetallic MCM-41 (Mobil Composition of Matter No. 41) and monometallic-SBA-16 catalysts (Santa Barbara Amorphous), resulting in three types of pyrolysis products: liquid products (bio-oil—BL_PYOIL_), gaseous products (syngas—BL_PYGAS_), and solid products (biochar—BL_PYCHAR_) [20]. 

The literature mentions that different catalysts have been explored in pyrolysis to enhance the quality of the resulting bio-oil [21]. Various studies have only focused on the incorporation of catalysts such as NiO, MoO_2_, and Co_3_O_4_ in a nitrogen atmosphere to reduce biochar formation and improve the properties of bio-oil [22]. When hydrogen was present, these three catalysts could enhance lignin degradation and boost bio-oil yield. Notably, Co_3_O_4_ significantly improved lignin catalytic pyrolysis in a H_2_/N_2_ atmosphere, resulting in a 26.38% increase in bio-oil yield [22]. Also, the pyrolysis of lignin from black liquor was improved with the addition of large-pore mesoporous materials (SBA-15) as catalysts and unilamellar mesoporous MFI nanosheets. Py-GC/MS was employed for analysis. It was observed that the quality of bio-oil was enhanced, with an increase in the quantities of aromatics and lighter phenolics, due to processes such as dehydration, decarbonylation, decarboxylation, and cracking, which occurred on the catalyst’s acid sites [23]. Taking all these factors into consideration, the present study focused on enhancing the quality of the resulting biochar in order to further valorise it as adsorbent material.

The introduction of the MCM-41-type mesoporous molecular sieve in 1992 represented a significant advancement in using these materials as supports for different catalytic species [24]. This material possessed high specific surface areas (around 1000 m^2^/g) and well-defined pore dimensions (2–30 nm), a relatively narrow pore size distribution, and a hexagonal arrangement of parallel mesopores. By controlling synthesis parameters (template selection, reaction temperature and time, or pH), high-quality MCM-41 can be achieved, improving characteristics like high specific surface area, pore volume, uniform pore size distribution, and thermal and chemical stability [21]. The addition of different metals, such as Al, Co, Cu, Fe, and Ni, has been demonstrated to enhance the catalytic properties of this nanomaterial [21,25]. MCM-41 can act as an effective support for dispersing Ni active sites due to its high specific surface area. However, it is unable to prevent the agglomeration of metallic nickel nanoparticles at elevated temperatures, as its pore diameters are smaller than those of the metal particles [26]. To overcome this issue, a cubic three-dimensional SBA-16-type silica can be effective at dispersing Ni nanoparticles [26]. SBA-16-type silica possesses robust pore walls, a high surface area, and excellent thermal and hydrothermal stability [27]. Additionally, as an all-silica material, MCM-41 has Si–OH groups on its surface that offer only a limited number of weak acid sites, restricting deoxidation and cracking capabilities during the pyrolysis process [28]. Consequently, several researchers have explored metal modification approaches, which can enhance both the structure and acidity of zeolite catalysts and improve catalytic performance and stability. Furthermore, this approach is becoming a promising method for upgrading bio-oil in biomass thermochemical processing. Also, when working with MCM-41, different metal was chosen to obtain the catalyst due to the fact that the Ni-MCM-41 did not have efficient catalytic effect on pyrolysis [28]. Furthermore, Cu-modified catalysts such as Cu/MCM-41 were reported to be efficient for upgrading bio-oil [29]. It was found that Cu incorporation effectively adjusted the acidity and textural properties of MCM-41, enhancing its deoxygenation capacity [29]. In this respect, a more efficient system (bimetallic Cu-Zn/MCM-41) was developed in this study, in which Zn was used as a promoter, in order to improve and overcome the above-mentioned limitations of MCM-41 catalysts.

The aim of this investigation was the removal of biochar resulting from the BL pyrolysis process. The necessity of removing biochar results from its high metal content and negative impact on the environment. BL_PYCHAR_ elimination, performed by valorisation, involves its transformation into an adsorbent material through chemical and thermal processes [30,31,32]. The final objective was CO_2_ removal and the identification of the adsorption capacity of the adsorbent materials prepared from the solid waste.

## 2. Materials and Methods

### 2.1. Adsorbents Preparation

The BL was procured from a pulp and paper factory (Drobeta-Turnu Severin, Romania), a kind of manufacturing that processes hard woods. 

The pyrolysis process was carried out in a fixed-bed reactor. The temperature was controlled from 300 °C to 450 °C, with a temperature gradient of 5 °C/min, in an inert atmosphere (N_2_/5.0). The flow rate of the inert gas, N_2_, was 100 mL/min. Three products resulted from the pyrolysis process: a gaseous product—BL_PYGAS_; a liquid product—BL_PYOIL_; and a solid product—BL_PYCHAR_. These can be considered alternative fuels. The first two products were the subject of the initial study [25]. 

The BL_PYCHAR_ was ground to a particle size <200 μm. After grinding, the chemical activation stage began. This was initiated by BL_PYCHAR_ treatment with 5 M HCl and KOH (Sigma Aldrich, Darmstadt, Germany) in a 1:1 ratio.

The catalysts used in the BL pyrolysis were two catalysts that were previously prepared in house (Cu-Zn-MCM-41 and Ni-SBA-16) [25]. These produced two types of biochar, denoted as 1a—BL_PYCHAR_Cu-Zn-MCM-41_ and 1b—BL_PYCHAR_Ni-SBA-16_. These two biochar materials constituted the raw materials for the development of the adsorbents. For the adsorbent’s development, HCl and KOH (Sigma Aldrich, Darmstadt, Germany) were used in the chemical activation process. Five adsorbents (Table 1) were developed from BL_PYCHAR_: two were produced after HCl treatment (2a—BL_PYCHARCu-Zn-MCM-41__HCl and 2b—BL_PYCHAR_Ni-SBA-16__HCl); two were produced after HCl and KOH treatment (3a BL_PYCHARCu-Zn-MCM-41__HCl+KOH (1:1) and 3b—BL_PYCHAR_Ni-SBA-16__HCl+KOH (1:1)); the fifth adsorbent was obtained after HCl and KOH treatment, followed by calcination (4a—BL_PYCHAR_Ni-SBA-16__HCl+KOH_c_). The calcination was carried out at a temperature of 750 °C, with a gradient of 10 °C/min, for 1 h in a N_2_ atmosphere at a flow rate of 100 mL/min.

**Figure 1:**
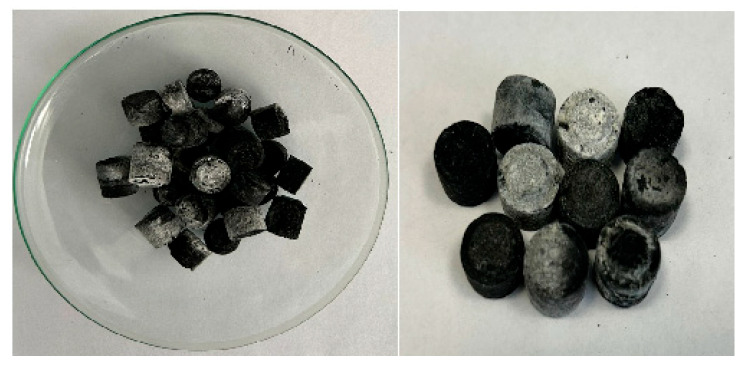
Black liquor char pellets.

After the thermal activation process, the materials obtained were subjected to a filtering process using filter paper type 389 (84 g/m^2^; particle retention 8–12 µm; thickness 0.19 mm) (Sartorious Stedim, Gottingen, Germany). The materials were washed with distilled water until pH 6, and then dried in an oven (Nahita 631, Auxilab, Navarra, Spain) without ventilation. The drying was carried out in two steps to avoid shocks on the pores of the formed materials: it was performed at 70 °C for 12 h and at 105 °, for 6 h. After the drying process, the obtained material was pelletized using a pelletizer (IKA, Staufen im Breisgau, Germany). The pellets had a mass of approximately 1 g (Figure 1). Then, they were subjected to a drying process at a temperature of 105 °C for 4 h.

### 2.2. Characterization of Biochar and Adsorbent Materials

The adsorbent materials were subjected to structural investigations such as elemental analysis, the determination of the heavy metal content, the determination of the specific surface area, morphological determinations, and FTIR analysis. The elemental analysis was performed using the Elemental Analyzer Flash 2000 (Thermo Scientific, Waltham, MA, USA), using the combustion method and the gas chromatographic method [26]. Heavy metal content was determined with NOVA A 300 Atomic Absorption Spectrophotometer (AAS) (Analytik Jena GmbH, Jena, Germany). The adsorbent materials were investigated via scanning electron microscopy with emission at a variable pressure. We used Field Emission Scanning Electron Microscope Variable Pressure—FESEM VP (CARL ZEISS, Oberkochen, Germany)—at a resolution of 0.8 nm at 30 kV and 2.5 nm at 30 kV in VP mode. We used the Brunauer–Emmett–Teller (BET) method for the measurement of the specific surfaces, and the analysis was performed using the Quantachrome Autosorb-IQ porosity equipment (Quantachrome Instruments, Boynton Beach, FL, USA.

The Fourier Transform Infrared Spectrometer Cary 630 ATR-FTIR (Agilent Technologies, Inc., Santa Clara, CA, USA) was used to determine the functional groups. The samples were first dried at 80 °C under vacuum conditions. The spectra were acquired with a attenuated total reflectance (ATR) module in the range 4000–400 cm° (32 scans, 8 cm^−1^ resolution, and 0.002 threshold). 

### 2.3. CO_2_ Adsorption Experiments

**Figure 2:**
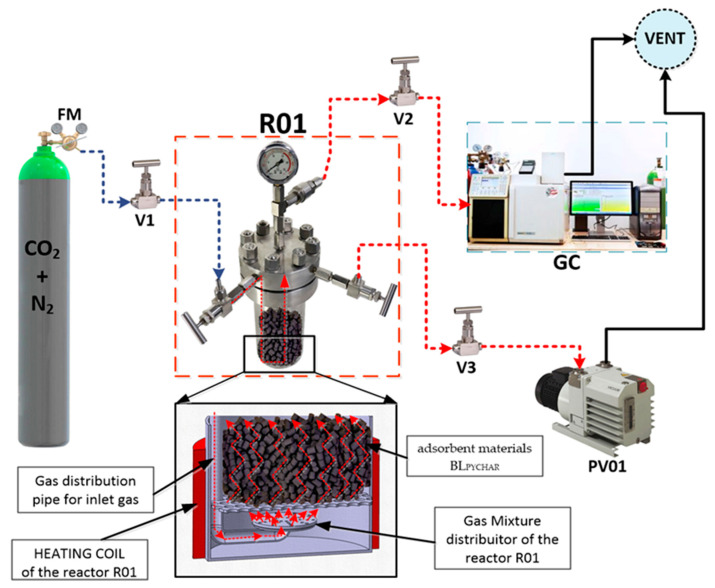
Flow sheet testing of adsorbent materials’ levels of BL_PYCHAR_: CO_2_ + N_2_—cylinder with test gas; FM—pressure and flow regulator; R01—reactor; Vx—manual valve; GC—gas chromatograph; PV01—vacuum pump.

The adsorbent materials were tested in an experimental setup, shown in Figure 2, with the aim of assessing CO_2_ removal and adsorption capacity.

To test the effectiveness of the adsorbent materials, a binary gas with the composition of 81% CO_2_ and 19% N_2_ was prepared and used. A cartridge was used. The adsorbent materials were inserted in the form of pellets at an approximate weight of 1 g/pellet with a total mass of 200 g. In order to observe in real time when the adsorbents reached saturation, the test installation was connected to a gas chromatograph with a TCD detector (GC Varian CP 3800, Varian Inc., Palo Alto, CA, USA). The conditions of the adsorbent materials were as follows: ambient temperature, 5 psi pressure, and 100 mL/min gas flow rate. The adsorption capacity of CO_2_, the yield, and the degree of recovery of adsorbent materials were calculated using the following equations [33]:

(1) $$a=\frac{Q\times p\left(C_{i}-C_{f}\right)\times t}{m}$$

where a = adsorption capacity, cm^3^/g; Q = the flow rate of the test gas passed over the adsorbent materials, cm^3^/s; p = adsorption pressure, bar; C_i_ = the initial CO_2_ concentration, %vol; C_f_ = the final CO_2_ concentration, %vol; t = time, s; m = adsorbent materials quantity from the reactor, g.

(2) $$R_{CO2}=\frac{{C_{e}}_{\left(CO2\right)}-{C_{i}}_{\left(CO2\right)}}{{C_{i}}_{\left(CO2\right)}}\times 100$$

where R_CO2_ = CO_2_ recovery, %vol; C_e(CO2)_ = average CO_2_ concentrations up to the rupture period at the exit from adsorbent materials, %vol; C_i(CO2)_ = initial CO_2_ concentration, %vol.

(3) $$\eta =\frac{C_{a}}{C_{i}}\times 100$$

where η = separation efficiency, %; C_a_ = the concentration of adsorbed CO_2_, %; C_i_ = the concentration of initial CO_2_, %.

## 3. Results and Discussion

Table 2 shows the elemental composition of the adsorbent materials developed from the residue obtained after the BL pyrolysis. The elemental analysis showed that the C level contributed the most to the biochar composition, followed by S, H, and N. This was due to the BL content in terms of cellulose, hemicellulose, and lignin [34]. After the treatment with 5 M HCl, a notable increase in the concentrations of C and S was observed in the BL_PYCHAR_ solid residues. Regarding the residue obtained following Cu-Zn-MCM-41 catalysis (group a), C and S concentrations reached 73.45% and 6.50%, respectively. Similarly, regarding the residue obtained from Ni-SBA-16 catalysis (group b), the C concentration rose to 77.04% and the S concentration rose to 6.21%. We also observed a decrease in the concentration of H after treatment with HCl, standing at 2.12% for 2a compared to the concentration seen for the BL_PYCHAR_ (6.51%). The data were in correlation with other biochars after being subjected to deashing methods from the literature [35]. 

After HCl treatment, the solid residues of both group a and group b suffered a decrease in metal content (Table 3), with levels between 18% (Cu) and 93% (Fe). A similar proportion was found for metals and elements in the second step of BL_PYCHAR_ transformation. The exception was seen for the initial Cu concentrations, which was 1139.21 mg/kg in the case of sample 1a and around 67.26 mg/kg in the case of 1b. It was previously demonstrated that, after deashing with a HCl solution, the amounts of transitional metal, as well as the levels of P, significantly decreased. This was probably the explanation behind the increase in the concentration of C and S [35].

**Figure 3:**
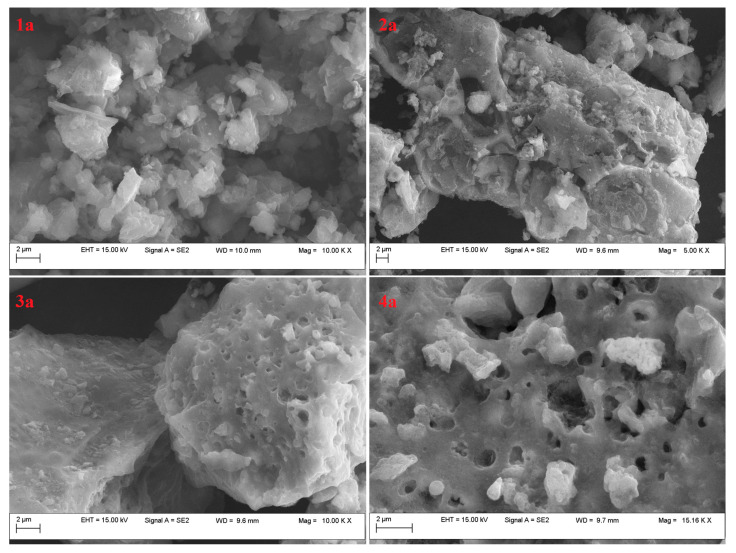
SEM images of the adsorbents resulted from BL_PYCHAR_ (Cu-Zn-MCM-41-catalysed pyrolysis) (group a).

**Figure 4:**
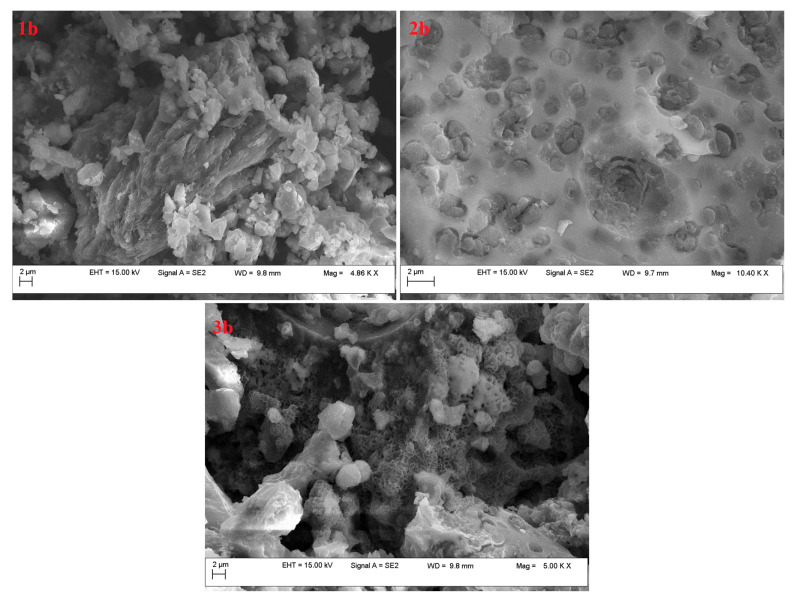
SEM images of the adsorbents taken from BL_PYCHAR_ (Ni-SBA-16-catalysed pyrolysis) (group b).

The SEM investigations (Figure 3 and Figure 4) indicated that all the developed adsorbents had porous structures. It should also be noted that they had an aggregated morphology [36]. The differences observed between the samples were a consequence of the preparation method. These observations suggested that BL_PYCHAR_-based adsorbents have different types of functional groups that can play the binding role for CO_2_ in different environments [37].

As for the calcined sample—4a, the grooves on the surface were destroyed, presenting a rougher surface (Figure 3(4a)). This was due to the decomposition of organic substances within the structure, and this was consistent with the literature [38].

After KOH treatment, clearer three-dimensional surface features were observed, results that were consistent with the literature (Figure 4) [39]. This may introduce the idea that these biochars should be more efficient in pollutant sequestration than HCl-based ones [36,37].

The surface chemistry and pore structure of the adsorbents essentially contribute to increasing the efficiency of the adsorption process. 

**Figure 5:**
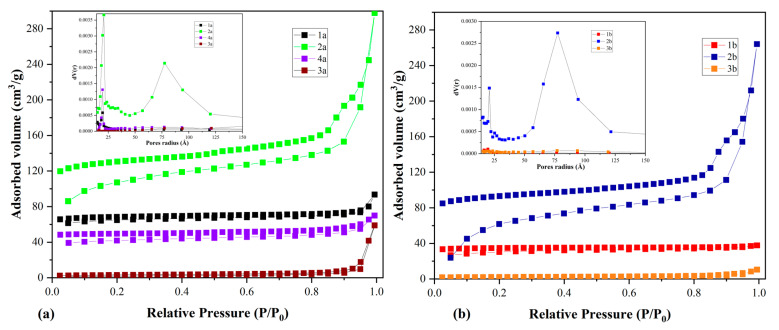
Adsorption isotherms and pore distribution of the BL_PYCHAR_-derived materials: (**a**) adsorbent—group a; (**b**) adsorbent—group b.

N_2_ adsorption–desorption isotherms are shown in Figure 5. The isotherms were classified as type II according to IUPAC as they were associated with a mesoporous structure, confirming the results obtained by the surface analysis. A mesoporous structure is highly beneficial for transporting CO_2_ from a gaseous mixture to the adsorbent surface, which increases the adsorption capacity [40]. Therefore, many functional groups from this type of material can provide effective active centres at the solid–gas surface interface, granting higher adsorption capacities in terms of CO_2_.

Specific surface area and microporosity are interconnected; the creation of numerous small micropores leads to an increased specific surface area. This larger surface area offers more active sites for CO_2_ adsorption via physical adsorption [41].

Analysing the samples obtained after HCl treatment, the highest specific surface area was reported for the sample 1a, 75.37 m^2^/g, although lignocellulosic biochars generally have low surface areas due to the nature of cellulose and hemicellulose (Table 4) [42]. BET investigations indicate that the sample 2b had a higher specific surface than the initial biochar, standing at 224.06 m^2^/g versus 54.29 m^2^/g, which was superior by comparison to the adsorbent 2a (Table 4). 

For all the obtained adsorbents, the pore volume was low. This is an attribute of the initial structure of cellulose, lignocellulose, and lignin, which are characterized by small amounts of pores or even blocked pores [43]. In addition, the materials showed small pore diameters, between 1.96 nm and 7.7 nm, indicating that these materials are mesoporous and may be suitable for use as adsorbents in the gas phase as they facilitate the diffusion of adsorbates into adsorbent structures. An interesting case was the sample 3a, which exhibited a higher pore diameter, around 30.3 nm, at the upper limit of the mesoporous interval at the same time as a drastic decrease in specific surface area. This was also in accordance with the morphology revealed via SEM (agglomerated particles and smother surface).

The adsorbent 2b stood out among all materials. It had an irregular morphology, as SEM investigation revealed (Figure 4(2b)), pore diameters of around 7.7 nm, and the highest surface area (224 m^2^/g), which represents an advantage in CO_2_ adsorption. As the isotherms showed, sample 2b showed a higher level of adsorption, with the pore volume reaching 0.28 cm^3^/g.

These findings were mainly correlated with the inorganic minerals blocking the pores of BL-based adsorbents being washed away, resulting in more exposed pores and improving the characteristics of the porous structure [35]. Moreover, inorganic minerals may obstruct direct contact between potassium hydroxide and the carbon skeleton to some extent, thus, potassium hydroxide can interact directly with the carbon structure, resulting in more efficient etching. The impact of potassium hydroxide activation becomes particularly noticeable after deashing with HCl. As a result, the specific surface area and micropore area of sample 2b were higher [35].

Earlier research indicated that adsorbents with a large surface area can exhibit a high capacity for CO_2_ capture. For example, when coffee grounds were used to produce biochar, the successful capture of CO_2_ was achieved at temperatures between 30 and 90 °C under a constant CO_2_ concentration, with a maximum adsorption capacity of 2.8 mmol/g at a BET surface area of 539 m^2^/g for the biochar [44].

**Figure 6:**
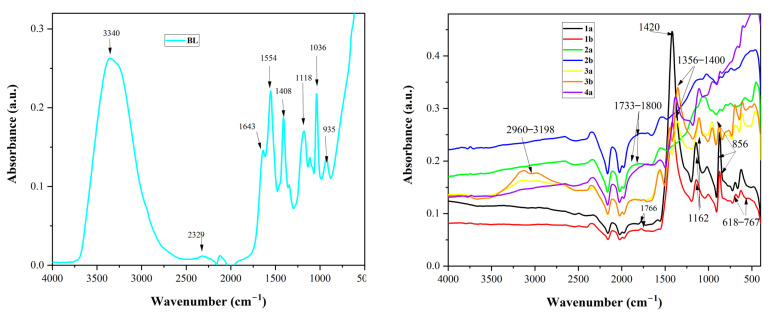
FT-IR spectra of BL (**left**) and the adsorbent materials derived from BL_PYCHAR_ (**right**).

Figure 6 and Table 5 present the FTIR spectra and peak assignment. For reasons of comparison, the black liquor (BL) spectrum was also introduced. Peaks with values below 1000 cm^−1^ characterized the deformation vibrations of the CH bonds associated with aromatic rings, while the absorption band around the value of 3340 cm^−1^ corresponded to the OH stretching vibration, indicating the presence of phenols, alcohols, or carboxylic acids in BL [45]. Also, the presence of a significant peak at 2329 cm^−1^ (representing the stretching vibration of the C-H bond in the methyl and methylene groups) was found. The peak at 1643 cm^−1^ indicated the vibrations of the aromatic backbone plus the C=O stretching vibration [45,46,47,48,49].

Compared to the FTIR spectrum of BL, the absorption peaks for BL_CHAR_ resulting from pyrolysis were much simpler. The band at 3340 cm^−1^, characterizing the -OH stretching vibration, disappeared after pyrolysis. This indicated the removal of alcohol groups from branched chains by pyrolysis (not phenols and carboxylic acids, as they mostly existed as phenolates and carboxylates in BL) [30]. The evolution of the peaks at about 1554 and 1643 cm^−1^ clearly indicated that the carbonyl and/or carboxyl groups were removed by pyrolysis. The removal was complete at temperatures above 450 °C. The peaks at 1420 cm^−1^ and 856 cm^−1^ were characteristic of absorptions due to potassium carbonate, revealing that its content increased upon treatment with potassium hydroxide [50]. The peaks of about 1162 cm^−1^ were assigned to the remaining C-O-C stretching of ester groups in cellulose and hemicellulose [51]. The three-peak group at 618–767 cm^−1^ in the FTIR spectra was attributed to residual aromatic C-H bands [51].

The peaks of KOH-activated adsorbent materials were stronger, indicating that the abundance of surface functional group species was higher. For example, the peak at 1766 cm^−1^ in the sample 1a, attributed to the C=O vibration, underwent a shift and transformation into a broad band in the activated materials at about 1733–1800 cm^−1^, indicating the intense presence of these groups on the surface. Also, the peaks at 1356–1400 cm^−1^ were assigned, as in the case of the starting material, to the C-H stretching vibrations of the CH_2_ and CH_3_ groups. The intensity of these peaks for the activated materials highlighted the increased rate of distribution of OH and CH_2_ structures. The biochar spectra of sample 3b showed alkyl bands around 2960 and 3198 cm^−1^, which were correlated with the hydrophobicity index of the remaining BL organic matter [51].

Table 6 represents the results obtained after testing the adsorbent material 2b, displaying the number of injections, the duration of the adsorption process, the evolution of CO_2_, the adsorption capacity of CO_2_, the yield, and the degree of recovery of these adsorbent materials. From all adsorbent materials, the sample 2b (2b—BL_PYCHAR_Ni-SBA-16__HCl) was chosen because of its high specific surface area (224.06 m^2^/g) and pore volume (0.28 cm^3^/g) and its medium diameter (7.7 nm). For comparison, the sample 3b (BL_PYCHAR_Ni-SBA-16__HCl+KOH (1:1)), with the lowest specific surface area (4.33 m^2^/g) and pore volume (0.01 cm^3^/g), was tested.

As can be seen in Table 6, the adsorption capacity of the adsorbent materials was tested on a mixture of gases. The saturation of the adsorbent materials could be assessed after 10 injections. The injection duration was 80 s, and the recovery of the CO_2_ concentration from the adsorbent materials was carried out using a vacuum pump at a pressure of 10^−2^ bar. The final adsorption capacity for sample 2b reached 18.57 cm^3^/g. The efficiency of the CO_2_ separation process from the gas mixture was 99.78% and the yield of the CO_2_ recovery process from the adsorbent materials was 62.98%. It was observed that, for the sample with the lowest specific surface area, which was 3b, the final adsorption capacity reached 17.59 cm^3^/g. In this process, the efficiency of the CO_2_ separation process from the gas mixture was 94.52% and the yield of the CO_2_ recovery process from the adsorbent materials was 40.45%. Although the difference between the adsorption capacity of the two samples was not very high, an increased recovery percentage was observed in the case of the sample with the highest surface area.

It can be stated that the CO_2_ adsorption process depended on parameters such as pressure and the adsorbent’s specific surface area. The tested adsorbent material, 2b, had a high specific surface area, showing a higher adsorption capacity (18.57 cm^3^/g) than a commercial adsorbent (activated carbon). It had a higher specific surface area (1470 m^2^/g), but a lower adsorption capacity, which started from 0.2 mmol/g (about 4.48 cm^3^/g) [52].

Various authors studied the impact of temperatures [53]. Temperature significantly affects the CO_2_ adsorption capacity, which is crucial for its capture following combustion. It was stated that the chosen temperature influenced the type of adsorption that took place, whether it was physisorption or chemisorption [53]. For example, at 30 °C, the adsorption capacity of commercial activated carbons reached 0.68 mmol CO_2_/g (about 15.23 cm^3^/g) adsorbent. The observed overall trend was that CO_2_ adsorption declined as temperature increased, falling to 0.17 mmol CO_2_/g (about 3.80 cm^3^/g) adsorbent at 70 °C [53]. These finding, together with the experimental results highlighted within the present study, align with the literature, which indicates that gas adsorption diminishes with increasing temperature [53]. As temperature increases at a constant flow rate, the kinetic energy of the gases also rises, resulting in reduced surface coverage of CO_2_. This pattern can be attributed to the exothermic nature of the adsorption process. Another parameter that can influence the adsorption capacity is the flow rate. The literature mentions that, in the case of using commercial activated carbons, the highest CO_2_ adsorption was observed at a flow rate of 30 mL/min and 30 °C [53]. Adsorption at flow rates up to 70 mL/min (30 °C—0.40 mmol CO_2_/g adsorbent and 70 °C—0.17 mmol CO_2_/g adsorbent) was lower than that achieved at 30 mL/min (30 °C—0.68 mmol CO_2_/g adsorbent and 70 °C—0.06 mmol CO_2_/g adsorbent) [53]. Reducing the gas inlet flow rate has been shown to increase contact time and enhance mass transfer between CO_2_ and the adsorbents. The motivation was that lower flow rates improved the retention time of CO_2_ molecules on the selected adsorbents within the packed bed adsorption column, leading to a higher amount of CO_2_ being adsorbed. At lower flow rates, the adsorbate (CO_2_) has more time to interact with the adsorbent, resulting in higher CO_2_ adsorption capacity [54].

It was demonstrated that activated carbon with a higher surface area had ab improved performance in terms of CO_2_ adsorption. However, some studies revealed that sugar cane-derived activated carbon exhibited improved adsorption sites compared to kaolinite or activated carbon–kaolinite composites at a temperature of 30 °C [53]. The kaolinite–activated carbon composite revealed an adsorption capacity of 0.42 mmol CO_2_/g (9.40 cm^3^/g), while kaolinite had the lowest capacity at 0.29 mmol CO_2_/g (6.49 cm^3^/g) [53]. The enhanced CO_2_ adsorption capacity of the activated carbon materials at low flow rates suggests an improved affinity for CO_2_ due to the presence of activated carbon. 

As was previously demonstrated, the amount of adsorbed CO_2_ showed a nearly linear increase with rising surface area, suggesting that this is a key factor affecting adsorption performance [55]. Also, no clear correlation was found between the volume of other pores and CO_2_ adsorption. In contrast, a relatively strong positive correlation was observed between micropore volume and CO_2_ adsorption [55]. These results indicated that the CO_2_ adsorption efficiency of a biochar may be primarily influenced by micropore size. The correlation between total pore volume and CO_2_ adsorption can be linked to the relationship between micropore volume and CO_2_ uptake [55]. 

Thus, it can be concluded that the development of micropores significantly impacts CO_2_ adsorption capacity. Consequently, it can be stated that surface area and micropore volume can serve as guidelines for obtaining materials with high CO_2_ adsorption characteristics.

## 4. Conclusions

Following the pyrolysis process of black liquor, under different experimental conditions, a solid BL_PYCHAR_ residue was obtained, which was further transformed into CO_2_-adsorbent materials. Seven types of adsorbent materials were developed, exhibiting relatively high specific surfaces and different pore diameters. The most efficient adsorbent material was 2b—BL_PYCHAR_Ni-SBA-16__HCl, which had the highest specific surface area of 224.06 m^2^/g, the highest pore volume of 0.28 cm^3^/g, and the highest pore diameter of around 7.7 nm compared to the other adsorbent materials treated with KOH, which had lower values. To test the efficiency of the 2b—BL_PYCHAR_Ni-SBA-16__HCl, a CO_2_ adsorption testing setup was used. The BL_PYCHAR_Ni-SBA-16__HCl adsorbent was tested on a gas mixture with a concentration of 81% CO_2_ and 19% N_2_, with saturation occurring after 10 injections. The adsorption capacity of the adsorbent material with the highest specific surface area was 18.57 cm^3^/g. It also had a separation efficiency of 99.78% and a recovery degree of 62.98%. The sample with the lowest specific surface area registered a final adsorption capacity of 17.59 cm^3^/g, with an efficiency of 94.52 % and a recovery of 40.45%. Although the difference between the adsorption capacity of the two samples was not so high, an increased recovery percent was observed in the case of the sample with the highest surface area. Therefore, the obtained material could be used as a potential candidate for CO_2_ capture and storage even at high concentrations. As a general conclusion, it can be stated that biochar resulting from waste valorisation is a promising candidate for CO_2_ capture materials, reducing anthropogenic CO_2_ emission and mitigating global warming. Although advancements were made in biochar obtention, more research is needed to produce adsorbents with increased adsorption capacity and long-term stability for scaling CO_2_ capture.

## Figures and Tables

**Table 1:** The nomenclature of the adsorbents obtained as a function of treatment.

Treatment	Adsorbent Material
HCl	2a—BL_PYCHARCu-Zn-MCM-41__HCl 2b—BL_PYCHAR_Ni-SBA-16__HCl
HCL and KOH	3a—BL_PYCHARCu-Zn-MCM-41__HCl+KOH (1:1) 3b—BL_PYCHAR_Ni-SBA-16__HCl+KOH (1:1)
HCl, KOH, calcination	4a—BL_PYCHAR_Ni-SBA-16__HCl+KOH_c_

**Table 2:** Elemental composition of BL_PYCHAR_ and resulted adsorbents.

Adsorbent Materials	H %	N %	S %	C %
BL_PYCHAR_	6.51	0.34	1.78	18.11
1a	0.93	0.22	2.10	34.96
1b	0.94	0.27	2.61	35.51
2a	2.12	0.47	6.50	73.45
2b	2.13	0.49	6.21	77.04
3a	1.87	0.29	1.04	36.62
3b	1.93	0.27	0.70	36.25
4a	1.17	0.30	0.96	37.06

**Table 3:** Heavy metals extracted from adsorbent materials derived from BL_PYCHAR_.

Adsorbent Materials	Pb mg/kg	Cu mg/kg	Fe mg/kg	Ni mg/kg	Mn mg/kg	Zn mg/kg	Ca mg/kg	Mg mg/kg
1a	<6.00	1139.21	2145.26	16.55	36.21	374.89	424.57	383.39
1b	<6.01	67.26	2089.27	49.56	32.13	81.52	159.73	258.83
2a	<6.02	929.40	138.98	<3.00	<4.00	27.96	66.57	27.38
2b	<6.03	69.46	75.37	15.91	<4.01	7.22	55.73	8.57
3a	<6.04	741.33	788.78	<3.00	<4.02	51.39	68.66	51.32
3b	<6.05	41.67	647.03	16.29	<4.03	18.08	68.99	50.24
4a	<6.06	1101.89	985.00	<3.00	4.09	82.72	62.86	67.19

**Table 4:** Textural properties of adsorbent materials derived from BL_PYCHAR_.

Adsorbent Materials	S_BET_ m^2^/g	V cm^3^/g	D_V_(r) Å
1a	75.37	0.04	19.68
2a	54.47	0.28	20.86
3a	6.36	0.09	303.78
4a	41.34	0.03	19.66
1b	54.29	0.01	19.65
2b	224.06	0.28	77.41
3b	4.33	0.01	76.98

**Table 5:** Peaks and bands identified in the BL spectrum.

Wavenumber (cm^−1^)	Assignment
935	C-H out of plan
1036–1118	C-O deformation from primary alcohols
1185	C-O vibration plus C=O and C-C from guaianyl and syringyl cores
1408	in-plane deformation of OH
1554	C-H vibration
1643	The vibration of the aromatic nucleus plus the C=O stretch
2329	C-H stretching from methyl and methylene groups
3340	O-H stretching from phenols, alcohols, and water

**Table 6:** CO_2_ adsorption evolution and efficiency.

Gas Mixture	Injection	CO_2_ Evolution vol%		Adsorption Capacity cm^3^/g		Separation Efficiency %		Recovery %	
		3b	2b	3b	2b	3b	2b	3b	2b
81 vol% CO_2_ balance N_2_	1	69.22	69.32	17.59	18.57	94.52	99.78	40.45	62.98
	2	69.01	58.13						
	3	67.33	49.77						
	4	62.47	40.12						
	5	60.66	31.03						
	6	59.05	22.75						
	7	42.44	15.47						
	8	29.91	9.33						
	9	17.81	3.78						
	10	4.44	0.18						

## Data Availability

Data are contained within the article.
